# Factors associated with social alienation in cancer patients: a structural equation modeling approach

**DOI:** 10.3389/fpsyg.2025.1721305

**Published:** 2025-12-10

**Authors:** Yaxuan Hu, Yemei He, Xiumei Liu, Shiyi Liao, Fang Xie, Ouying Chen, Jie Zhang

**Affiliations:** 1 School of Nursing, Hunan University of Chinese Medicine, Changsha, China; 2 The First Hospital of Hunan University of Chinese Medicine, Changsha, China

**Keywords:** oncology, cancer, social alienation, structural equation modeling, factors

## Abstract

**Introduction:**

Social alienation is an important issue for patients with cancer. However, the interrelationships among factors influencing social alienation in cancer patients have not been sufficiently investigated. The study aimed to clarify the relationships among social alienation, illness perception, fear of cancer progression, and perceived social support in patients with cancer.

**Methods:**

A cross-sectional descriptive survey was conducted with 244 cancer patients recruited through convenience sampling from a tertiary hospital in Changsha, China. Data were collected using the General Information Questionnaire, Brief Illness Perception Questionnaire, General Alienation Scale, Fear of Progression Questionnaire-Short Form, and Perceived Social Support Scale.

**Results:**

The findings show that the mean social alienation score among cancer patients was 33.11 ± 7.95. Model fit indices indicated a good fit. Illness perception and perceived social support have a direct and significant negative impact on social alienation, with path coefficients of −0.19 and −0.25, respectively. Fear of cancer progression has a direct and significant positive effect on social alienation, with a path coefficient of 0.45. Additionally, the results of the mediation analysis indicate that illness perception indirectly influences social alienation through its effects on fear of cancer progression and perceived social support; employment status indirectly influences social alienation through illness perception; disease stage indirectly influences social alienation through illness perception and fear of cancer progression.

**Conclusion:**

This suggests that illness perception, fear of cancer progression, and perceived social support are key factors influencing social alienation in cancer patients. These factors exerted both direct and indirect effects on each other and on social alienation.

## 1 Introduction

Cancer has been one of the most significant global public health challenges in the 21st century. According to the International Agency for Research on Cancer, there were approximately 19.98 million new cancer cases worldwide in 2022, including about 4.83 million in China (Wang et al., 2024). The report Cancer Statistics, 2025 indicates that the 5-year relative survival rate for cancer worldwide during the period 2014–2020 was approximately 69% (Siegel et al., 2025). However, cancer and its treatment exert complex influences on patients’ physical, psychological, and social functioning.

Social alienation refers to a state of deficiency or detachment within an individual’s social network, encompassing both subjective feelings of loneliness and objective social withdrawal (Umberson and Donnelly, 2023). And it is closely linked to psychological status and health risks. The side effects of treatment, economic burdens, and disruptions in social relationships gradually undermine the social functioning of cancer patients. A cohort study reported that 27∼30% of young cancer patients experienced social alienation (Li et al., 2024). Such alienation contributes to symptoms of depression and anxiety, and in severe cases, suicidal ideation (Li et al., 2024; Ernst et al., 2021). Moreover, social alienation may promote tumor growth, reshape the tumor immune microenvironment, and lead to immunosuppression, thereby increasing cancer-specific mortality (Miaskowski et al., 2021; Trachtenberg et al., 2024; Zhao et al., 2024). Surveys indicate that cancer patients have a strong need for social support, yet this need is often neglected (Guo and Cui, 2025).

Illness perception refers to an individual’s subjective understanding and evaluation of various aspects of a disease, such as its causes, symptoms, and consequences (Striberger et al., 2020). Studies have shown that negative illness perceptions are significantly associated with poorer psychological outcomes and quality of life (Jabbarian et al., 2021). Among breast cancer patients, negative illness perceptions impair disease management and reduce quality of life (Li et al., 2025). Furthermore, negative perceptions of illness can heighten patients’ anxiety and fear regarding their condition, potentially leading to self-stigmatization, increased social avoidance, social isolation, and the formation of a vicious cycle (Miaskowski et al., 2021; Fox et al., 2023).

Fear of disease progression refers to patients’ persistent fear of disease worsening or recurrence, often manifested as hypervigilance toward physical symptoms and avoidant behaviors (Berg et al., 2011). Among cancer patients, fear of disease progression typically manifests as concern about cancer worsening, and this fear is closely associated with increased risks of anxiety and depression (Sharpe et al., 2022). Severe distress related to fear of progression may impair cognitive functioning and directly reduce quality of life. A study on hemodialysis patients found that fear of disease progression mediated the relationship between illness perception and social alienation (Zhu et al., 2024).

Perceived social support refers to an individual’s awareness of receiving psychological and material support from family, friends, and broader social networks (Jiang et al., 2021). Studies have demonstrated that social support significantly influences patients’ quality of life. Moreover, social support in cancer care is modifiable: supportive care interventions can improve quality of life (Li et al., 2024). A cross-national study further revealed that higher perceived social support mitigates fear of illness and reduces social alienation (Kim and Jung, 2020).

The Multi-factor Model of Psychological Stress, proposed by Jiang (2014) through long-term empirical research, incorporates multiple interacting systems, including life events, cognitive appraisal, coping strategies, social support, personality traits, and psychosomatic responses, with cognition as the central element. Existing studies suggest that illness perception, fear of disease progression, and perceived social support are closely related to social alienation (Zhu et al., 2024; Kim and Jung, 2020). Given that our research focuses on cancer patients, we will specifically define the fear of disease progression as the fear of cancer progression. Specifically, illness perception represents cancer patients’ direct cognition of disease, fear of progression constitutes a psychological reaction based on that cognition, and perceived social support represents the social dimension. Therefore, grounded in this theoretical framework, the present study incorporated these three variables into a structural equation model (SEM) for analysis.

Although social alienation plays a critical role in psychological stress and mental health among cancer patients, substantial gaps remain in understanding its interrelationships with illness perception, fear of cancer progression, and perceived social support, as well as their implications for clinical practice. Therefore, based on a literature review, we developed a hypothetical model incorporating illness perception, fear of cancer progression, perceived social support, and social alienation. We hypothesize that Illness Perception will negatively affect Social Alienation, and this relationship will be mediated by Fear of Cancer Progression and Perceived Social Support. Furthermore, Fear of Cancer Progression will directly positively influence Social Alienation, while Perceived Social Support will directly negatively influence Social Alienation. The primary aim of this study was to test this model using structural equation modeling, in order to clarify the relationships among these factors in cancer patients and provide evidence for the development of targeted interventions.

## 2 Materials and methods

### 2.1 Research question

Based on a multi-factor model of psychological stress, this study aims to examine the negative impact of Illness Perception on Social Alienation, as well as the mediating roles of Fear of Cancer Progression and Perceived Social Support. Therefore, the following research questions are proposed:

- Research Question 1: Does Illness Perception have a negative impact on Social Alienation ?
- Research Question 2: Is the effect of Illness Perception on Social Alienation mediated by Fear of Cancer Progression and Perceived Social Support?
- Research Question 3: How do Fear of Cancer Progression and Perceived Social Support affect Social Alienation?

### 2.2 Design and sample

This study is a descriptive cross-sectional study. We used convenience sampling to recruit cancer patients from a tertiary-level hospital in Changsha, China. Inclusion criteria: (1) Patients diagnosed with cancer by cytology or histology; (2) Age ≥ 18 years; (3) Patients who had completed initial anticancer treatments such as surgery or radiotherapy/chemotherapy and were in the follow-up period; (4) Patients who could read or write; (5) Patients who were aware of their condition and consented to participate in this study. Exclusion criteria: (1) Cancer metastasis or recurrence, given that patients with metastatic or recurrent cancer present with more severe disease, heavier treatment burdens, and greater psychological stress, such patients were excluded from the study to ensure clearer and more targeted research findings; (2) Concurrent severe chronic diseases; (3) History of mental illness or communication barriers.

Typically, a median sample size of 200 is recommended for SEM analysis (Kline, 2011). An *a priori* sample size calculator for SEM was applied, which is popular and publicly designed for calculating SEM sample sizes^1^ (Soper, 2015). The minimum sample size with a moderate effect (0.3), at a power value of 0.95, including 4 latent and 20 observed variables (all observed indicators and sociodemographic variables), and with an α of 0.05 was calculated as 207. Considering 10% dropout, we chose a minimum sample size of 227.

### 2.3 Ethical approval

This study was approved by the Institutional Review Board of the School of Nursing of Hunan University of Chinese Medicine. All eligible participants provided informed consent before completing the questionnaire. They were informed of their rights in the study, including their right to withdraw from the study at any time.

### 2.4 Measurements

#### 2.4.1 General Information Questionnaire

A self-made general survey questionnaire was used to collect demographic data on patients, including age, gender, educational attainment, marital status, employment status, monthly household income, medical expenses, disease stage, and comorbidities.

#### 2.4.2 Brief Illness Perception Questionnaire

The Brief Illness Perception Questionnaire (BIQP) is used to measure patients’ self-perception of their illness. The questionnaire was developed by Broadbent et al. (2005) and translated into Chinese by Ma et al. (2015). It consists of 9 items (impact of illness, course of illness, personal control, treatment control, symptoms experienced, concern about illness, understanding of illness, emotions, perceived cause of illness) and 3 dimensions (illness cognition, emotions, understanding ability). The 9th item is an open-ended question, while the remaining 8 items are scored on a 0–10 point scale. For quantitative data analysis, this study focuses on the scores of the first 8 items to assess patients’ illness perception, with a total score of 80 points. Higher scores indicate that patients perceive their disease symptoms as more severe. The Cronbach’s α for this scale in this study is 0.929.

#### 2.4.3 General Alienation Scale

The General Alienation Scale (GAS) is used to assess patients’ feelings of social alienation. The scale was developed by Pattison (1978) and translated into Chinese by Chen et al. (2015) in 2015. The scale consists of 15 items across 4 dimensions (alienation from others, feelings of powerlessness, self-alienation, and feelings of meaninglessness), using a 4-point Likert scale, with scores ranging from 1 to 4, corresponding to “strongly disagree” to “strongly agree.” A higher total score indicates a higher degree of social alienation in patients. The Cronbach’s α for this scale in the present study was 0.907.

#### 2.4.4 Fear of Progression Questionnaire Short Form

The Fear of Progression Questionnaire Short Form (FoP-Q-SF) was developed by Mehnert et al. (2009) based on the FoP-Q, and translated into Chinese by Wu et al. (2015). This study used the questionnaire to assess patients’ fear of cancer progression. The questionnaire consists of 12 items, including 2 dimensions: physiological health factors and social and family factors. A 5-point Likert scale was used, with scores ranging from 1 to 5, representing “never” to “always.” A higher total score indicates greater fear of cancer progression. The Cronbach’s α for this questionnaire in this study was 0.940.

#### 2.4.5 Perceived Social Support Scale

The Perceived Social Support Scale (PSSS) is used to measure patients’ perceptions of the level of support they receive from family, friends, and other individuals. Originally developed by Zimet et al. (1988), it was revised into Chinese by Jiang (1999). The scale consists of three dimensions and 12 items, using a 7-point scoring method, with scores ranging from 1 (strongly disagree) to 7 (strongly agree). A higher total score indicates a higher level of perceived social support. The Cronbach’s α for this questionnaire in this study was 0.958.

### 2.5 Data collection

Convenience sampling was used, and the data were collected from a tertiary-level hospital in Changsha between May 2024 and June 2025. The researchers first contacted the head nurse of the hospital’s oncology department and explained the purpose and methods of the study in detail. After obtaining the head nurse’s consent, two researchers entered the ward and collected data through the “QuestionStar” electronic questionnaire platform. Based on the time researchers spent completing the questionnaire in advance, we set 5 min as the average completion time. A total of 245 questionnaires were distributed, and one patient declined to participate in the study. Ultimately, 244 valid questionnaires were collected.

### 2.6 Data analysis

Descriptive and correlation analyses were performed using SPSS 27.0 software. Quantitative data were expressed as $\bar{x}\,s$, and categorical data were expressed as frequencies and proportions. Pearson correlation analysis was used to describe the correlation between two variables. Independent samples *t*-tests and analysis of variance (ANOVA) were used to analyze the effects of sociodemographic factors on intergroup differences in variable scores. Additionally, we incorporate sociodemographic factors identified in Table 1 as exogenous observed variables into the SEM to predict latent variables (Illness Perception, Fear of Cancer Progression, Social Alienation and Perceived Social Support). AMOS 26.0 was used to construct the SEM. The fit index, incremental fit index, normality fit index, and goodness-of-fit index were used as validation indicators for the SEM. If all index values were >0.90, the model was considered to have a good fit. A root mean square error of approximation (RMSEA) <0.05 indicated good fit; <0.08 indicated the fit results were within an acceptable range. Approximate maximum likelihood estimation was used to estimate regression coefficients and effect sizes. Based on the significance test results of path coefficients and model fit indices, the model was revised to optimize its parsimony. *P* < 0.05 indicates statistically significant differences (Brown, 2006; Hu and Bentler, 1999).

**TABLE 1:** Relationship between sociodemographic characteristics and variable scores (*n* = 244).

Variables	N (%)	BIQP	FoP-Q-SF	PSSS	GAS
**Age (years)**					
18∼30	23 (9.4)	42.04 ± 19.75	38.87 ± 10.06	57.83 ± 17.98	32.61 ± 9.05
31∼40	15 (6.1)	56.40 ± 15.89*	37.13 ± 10.59	69.40 ± 16.30	32.13 ± 8.89
41∼50	26 (10.6)	46.85 ± 17.64	33.19 ± 9.36	63.81 ± 14.55	34.92 ± 8.05
51∼60	79 (32.4)	53.89 ± 14.46	35.03 ± 11.57	61.94 ± 13.55	33.10 ± 8.10
61∼70	64 (26.2)	55.88 ± 10.65	35.08 ± 8.31	66.73 ± 12.80	33.89 ± 7.48
≥71	37 (15.2)	54.05 ± 14.92	31.27 ± 11.37	64.24 ± 12.00	31.19 ± 7.34
**Religious belief**					
None	222 (91.0)	52.53 ± 15.27	34.53 ± 10.63	63.86 ± 13.93	32.91 ± 8.05
Yes	22 (9.0)	54.68 ± 10.86*	37.14 ± 7.93	63.36 ± 15.63	35.09 ± 6.87
**Educational level**					
Primary school or below	32 (13.1)	57.25 ± 9.48*	33.47 ± 7.27	62.63 ± 13.84	31.66 ± 7.44
Middle school	83 (34.0)	51.28 ± 13.50	33.64 ± 11.04	62.49 ± 13.64	34.75 ± 7.69
High school or technical secondary school	64 (26.2)	56.34 ± 14.89	35.17 ± 10.22	65.75 ± 13.80	32.03 ± 6.91
College	53 (21.7)	48.21 ± 18.16	35.79 ± 10.90	64.21 ± 15.87	32.57 ± 9.16
Master’s degree or above	12 (4.9)	51.25 ± 15.14	39.33 ± 11.99	64.08 ± 14.00	33.75 ± 9.94
**Marital status**					
Unmarried	23 (9.4)	43.96 ± 17.32	39.43 ± 9.96	58.26 ± 14.76	33.87 ± 8.74
Married	205 (84.0)	53.25 ± 14.70	34.29 ± 10.45	64.68 ± 14.05	33.08 ± 7.93
Other	16 (6.6)	58.50 ± 8.55*	34.19 ± 9.76	60.75 ± 11.52	32.38 ± 7.64
**Employment status**					
Employed	50 (20.5)	47.20 ± 18.87	36.36 ± 11.46*	63.22 ± 15.37	32.40 ± 8.31
Unemployed	36 (14.8)	43.61 ± 17.75	30.81 ± 11.26	62.61 ± 16.97	30.92 ± 8.28
Other	158 (64.8)	56.54 ± 10.95*	35.16 ± 9.72	64.28 ± 12.94*	33.83 ± 7.71
**Monthly household income (CNY)**					
≤2000	43 (17.6)	54.88 ± 12.81	35.26 ± 10.36	62.40 ± 15.43	34.30 ± 10.36
2000–5000	79 (32.4)	50.62 ± 16.28*	34.77 ± 11.28	61.48 ± 13.45	34.29 ± 7.57
5000–8000	68 (17.9)	49.78 ± 16.09	34.09 ± 10.38	64.53 ± 14.38	31.87 ± 7.08
≥8000	54 (22.1)	57.78 ± 11.24	35.22 ± 9.45	67.46 ± 12.90	31.98 ± 7.17
**Disease stage**					
I	56 (23.0)	48.98 ± 18.01	38.02 ± 11.34*	63.16 ± 17.17	33.68 ± 10.30
II	58 (23.8)	50.43 ± 12.86	35.29 ± 9.07	61.17 ± 12.79	34.05 ± 7.33
III	63 (25.8)	49.94 ± 16.24	29.68 ± 10.06	62.19 ± 13.28	32.75 ± 6.89
IV	67 (27.5)	60.45 ± 8.66*	36.37 ± 9.53*	68.18 ± 12.13*	32.15 ± 7.20
**Medical burden**					
No burden	18 (7.4)	52.00 ± 19.24	30.67 ± 12.80	65.61 ± 20.19	27.61 ± 9.61
Minimal burden	64 (26.2)	49.66 ± 18.02	32.27 ± 10.07	65.64 ± 13.50	32.56 ± 6.73
Moderate burden	117 (48.0)	52.87 ± 12.80	36.18 ± 10.50	63.39 ± 12.50	34.22 ± 8.12*
Heavy burden	45 (18.4)	56.98 ± 12.55	36.29 ± 8.83*	61.60 ± 15.80	33.18 ± 7.74

*P < 0.05. BIQP, Brief Illness Perception Questionnaire; FoP-Q-SF, Fear of Progression Questionnaire Short Form; PSSS, Perceived Social Support Scale; GAS, General Alienation Scale.

## 3 Results

### 3.1 Sample characteristics and its difference among variable scores in the model

A total of 245 patients was recruited for the survey, and 244 cases were included in the statistical analysis (1 patient refused to complete the questionnaire during the survey). Age, religious beliefs, marital status, educational attainment, employment status, monthly household income, disease stage, and medical burden influenced one or more latent variables (Table 1). Therefore, these sociodemographic variables were included as covariates in the model.

### 3.2 Measured variables and their relationships

The mean total scores for BIQP, FoP-Q-SF, PSSS, and GAS among cancer patients were 52.72 ± 14.92, 34.77 ± 10.43, 63.82 ± 14.06, and 33.11 ± 7.96, respectively. Statistical data for each dimension are presented in Table 2.

**TABLE 2:** Descriptive statistics of measured variables (*n* = 244).

Variables	Mean	SD	Median	Range
BIQP	52.72	14.92	54.50	0.00–80.00
Illness representation	32.67	9.80	34.00	0.00–50.00
Emotions	13.47	4.09	14.00	0.00–20.00
Comprehensibility	6.58	2.13	7.00	0.00–10.00
FoP-Q-SF	34.77	10.43	36.00	12.00–60.00
Fear of physical health	18.57	5.44	18.00	6.00–30.00
Fear of social and family consequences	16.20	5.64	16.00	6.00–30.00
PSSS	63.82	14.06	65.00	12.00–84.00
Family support	22.60	4.64	23.00	4.00–28.00
Friend support	20.92	5.38	22.00	4.00–28.00
Other support	20.30	5.38	20.00	4.00–28.00
GAS	33.11	7.96	33.00	15.00–60.00
Alienation from others	9.86	3.31	10.00	5.00–20.00
Powerlessness	9.84	2.05	10.00	4.00–16.00
Self-alienation	6.71	2.07	7.00	3.00–12.00
Meaninglessness	6.69	1.64	7.00	3.00–12.00

Patients’ perception of the disease and fear of cancer progression were significantly positively correlated with perceived levels of social support; patients’ feelings of social isolation were significantly positively correlated with fear of cancer progression and significantly negatively correlated with perceived levels of social support. Additionally, there was no significant relationship between patients’ perception of the disease and feelings of social isolation, or between fear of cancer progression and perceived levels of social support (Table 3).

**TABLE 3:** Correlations among the measured variables (*n* = 244).

Variables	BIQP	FoP-Q-SF	PSSS	GAS
BIQP	1.000	–	–	–
FoP-Q-SF	0.462^a^	1.000	–	–
PSSS	0.251^a^	0.031	1.000	–
GAS	0.069	0.356^a^	−0.256^a^	1.000

^a^*P* < 0.01.

#### 3.3 SEM of measured variables

**FIGURE 1:**
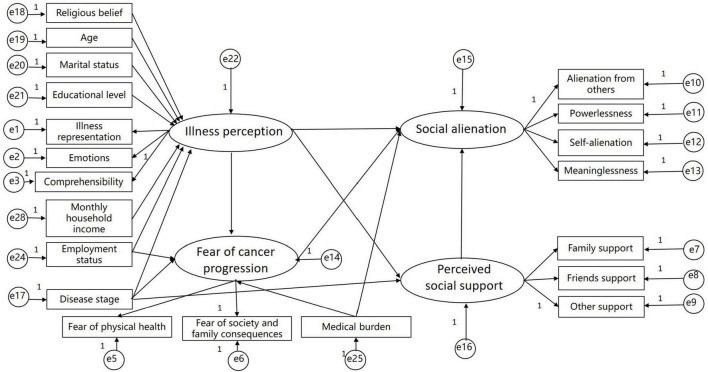
The hypothetical model.

**FIGURE 2:**
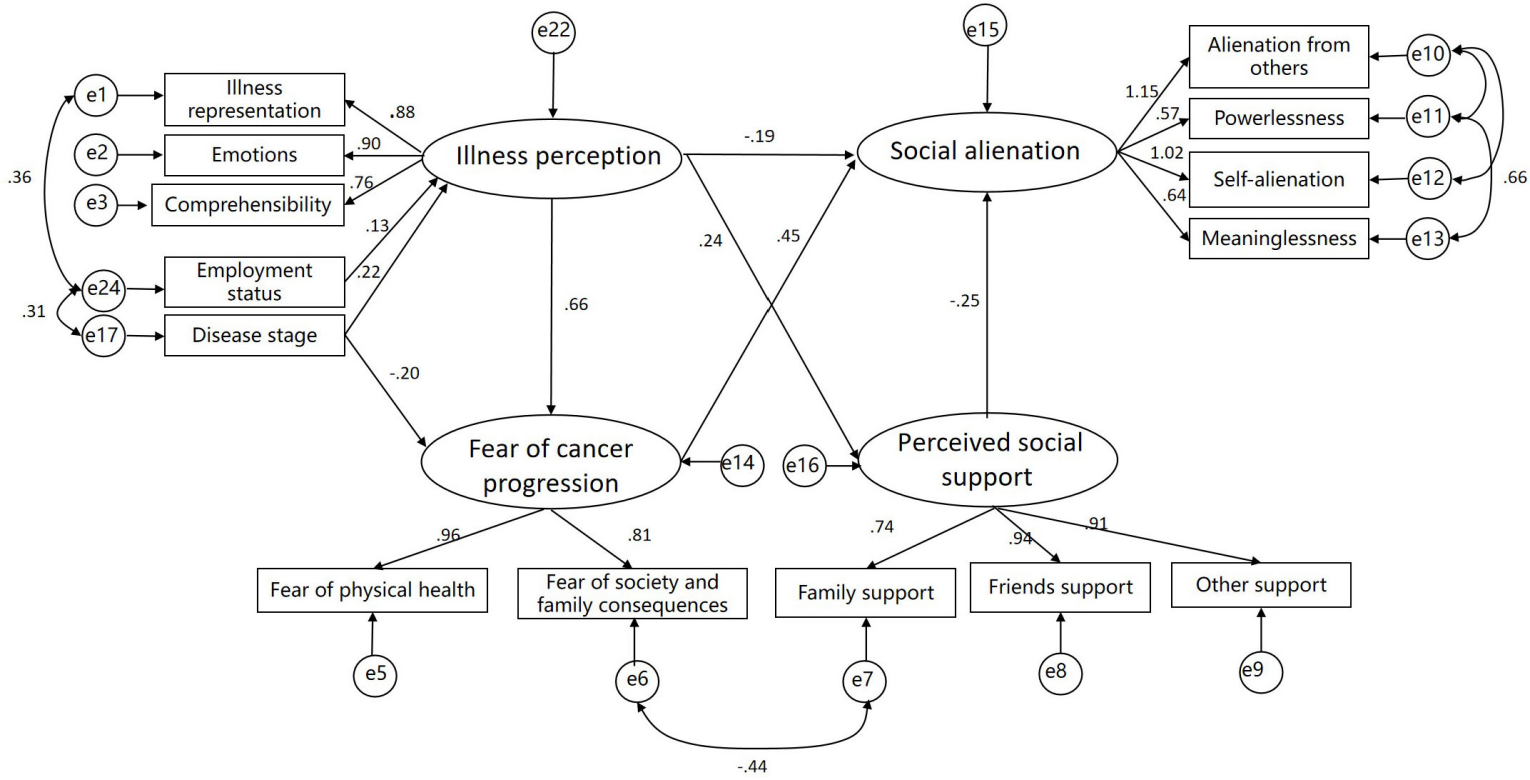
The modified model.

The hypothetical model (Figure 1) does not fit the data well, we modified the model (Figure 2) based on the correction index and deleted paths with small standardized coefficients (absolute value < 0.1). Therefore, the following paths were removed: Religious belief→Illness perception, Age→Illness perception, Marital status→Illness perception, Educational level→Illness perception, Monthly household income→Illness perception, Disease stage→Perceived social support. Although the modified model showed significant differences (*P* < 0.05), other indicators showed that it fit well (Table 4).

**TABLE 4:** SEM goodness-of-fit evaluation results.

Model	CMIN(P)	DF	CMIN/DF	GFI	NFI	IFI	CFI	RMSEA
Reference	>0.05		<3	0.9–1	0.9–1	0.91	0.9–1	<0.08
Hypothetical	781.084 (0.000)	161	4.851	0.710	0.713	0.758	0.755	0.126
Modified	186.466 (0.000)	65	2.869	0.901	0.918	0.945	0.945	0.088

Structural equation model indicates that illness perception and perceived social support have a direct and significant negative impact on social alienation. Although correlation analysis revealed no significant association between patients’ disease cognition and social isolation (Table 3), SEM uncovered a direct negative relationship between the two (Table 5). This discrepancy likely stems from other complex pathways obscuring the true association between these variables in the correlation analysis. Therefore, SEM must be employed to explore the intricate mechanisms underlying these variables. Fear of cancer progression has a direct and significant positive effect on social alienation (Table 5). Additionally, the results of the mediation analysis indicate that illness perception indirectly influences social alienation through its effects on fear of cancer progression and perceived social support; employment status indirectly influences social alienation through illness perception; disease stage indirectly influences social alienation through illness perception and fear of cancer progression (Table 6).

**TABLE 5:** SEM path coefficients.

Pathway	Non-standardized coefficients	Standardized coefficients	SEs	Critical ratio	*P*
Social alienation ← Illness perception	−0.436	−0.187	0.154	−2.834	0.005
Social alienation ← Fear of cancer progression	0.367	0.449	0.055	6.690	<0.001
Social alienation ← Perceived social support	−0.191	−0.247	0.038	−4.973	<0.001
Perceived social support ← Illness perception	0.740	0.245	0.210	3.527	<0.001
Fear of cancer progression ← Illness perception	1.885	0.659	0.223	8.441	<0.001

**TABLE 6:** Standardized direct, indirect, and total effects for the modified model.

Endogenous variables	Exogenous variables	Standardized direct effects	Standardized indirect effects	Standardized total effects
Social alienation	Illness perception	−0.187	0.236	0.049
	Fear of cancer progression	0.449	0	0.449
	Perceived social support	−0.247	0	−0.247
	Disease stage	0	−0.780	−0.780
	Employment status	0	0.006	0.006
Perceived social support	Illness perception	0.245	0	0.245
Fear of cancer progression	Illness perception	0.659	0	0.659

## 4 Discussion

### 4.1 Current state of social alienation

In this survey, the mean social alienation score among cancer patients was 33.11 ± 7.96, indicating a medium-to-high level. This result is consistent with findings from studies on other chronic diseases in China (Xu et al., 2024; Li et al., 2022). Research conducted in other countries has shown that more than 26.6% of patients experience psychosocial distress (Baye et al., 2023). Collectively, these studies suggest that patients with cancer and other chronic illnesses commonly experience social alienation. The underlying reasons may be related to disease itself and treatment-related side effects, which restrict physical functioning and alter appearance, thereby reducing patients’ social capacity and willingness (Fox et al., 2023). In addition, cancer patients often suffer from elevated psychological stress and poor mental health, both of which further exacerbate social alienation (Li et al., 2024).

### 4.2 SEM analysis

This study employed SEM to examine the relationships among social alienation, illness perception, fear of cancer progression, and perceived social support in Chinese cancer patients. Our findings contribute to a deeper understanding of the pathways influencing social alienation and support the development of interventions targeting social alienation in this population.

#### 4.2.1 Illness perception

This study found that illness perception had a direct impact on social alienation. Negative cognitions regarding disease severity, treatment side effects, and prognosis increased patients’ psychological burden (Kim et al., 2022). It reduced their motivation to socialize, resulting in objective social withdrawal behaviors and subjective feelings of loneliness. Additionally, illness perception indirectly influenced social alienation via fear of cancer progression. Negative cognitions not only directly led patients to reduce social contact but also heightened fear of progression, producing psychological stress (Sharpe et al., 2023). Such fear further encouraged avoidance of potentially health-threatening social situations, thereby intensifying social alienation. Psychological stress can activate the neuroendocrine system, increase inflammatory cytokine levels, and subsequently impair brain function, which reinforces social withdrawal (Kim et al., 2022). Evidence suggests that professional psychological interventions helping patients distinguish realistic risks from excessive worries may foster more accurate illness perceptions, enhance quality of life, and reduce social alienation (Barello et al., 2022). Hence, empowerment education focusing on emotion regulation and stress relief may help patients cope positively with negative emotions.

Moreover, this study found that perceived social support mitigated the level of social alienation. Perceived social support is a core protective factor that can improve patients’ social connectedness. Specifically, perceived support is negatively associated with limbic system activity, reducing negative emotional responses and enabling patients to feel “respected” and “accepted,” thereby decreasing social avoidance and promoting active social engagement (Borgers et al., 2024; Liu et al., 2021). Illness perception also exerted an indirect effect on social alienation through perceived social support. Evidence indicates that positive illness perceptions are independently associated with higher levels of perceived social support (Mathieu et al., 2024). Greater perceived support buffers the impact of negative illness cognitions and emotions, reducing loneliness, whereas insufficient support amplifies these effects and aggravates alienation. Healthcare providers can act as bridges between patients and social support systems by encouraging open communication with family and friends and organizing online/offline peer-support groups to facilitate emotional support.

#### 4.2.2 Disease stage

This study revealed that disease stage indirectly affected social alienation through illness perception or fear of cancer progression. As an objective clinical indicator, disease stage directly shapes patients’ subjective perceptions of disease severity and prognosis. A higher tumor stage implies greater disease severity and poorer prognosis, thereby heightening patients’ sense of threat, including concerns about survival rates and the emergence of negative emotions such as depression and anxiety (Liu et al., 2023). It also directly intensifies fear of cancer progression, ultimately leading to social withdrawal and increased social alienation.

#### 4.2.3 Employment status

This study demonstrated that employment status indirectly influenced social alienation through illness perception. Employment itself represents an important form of social support (Leep Hunderfund et al., 2022). Cancer patients often face substantial financial burdens, and work serves as a crucial source of income. Job loss sharply reduces income, creating financial stress, while an unsupportive work environment may foster workplace exclusion (Carlson et al., 2022). Patients often attribute these difficulties to their illness, thereby forming erroneous negative perceptions of their condition. They view the disease as a devastating economic event and a core factor threatening their social role positioning, believing it robs them of normal working capacity, diminishes their self-worth, and lowers their social standing. Consequently, they adopt avoidant coping behaviors and reduce social engagement. Healthcare providers can help link patients with community services, charitable foundations, and government subsidies to alleviate financial stress.

### 4.3 Limitations

This study has three limitations: (1) The sample was drawn from a single hospital in Changsha, China, which may limit generalizability to all cancer patients. (2) The cross-sectional design restricts causal inference among variables. (3) Convenience sampling may introduce selection bias, thereby limiting the validity of research findings to some extent. (4) The SEM does not encompass all potential factors that may influence social alienation among cancer patients, such as common psychological states like depression, anxiety, and stress. In the future, we will incorporate patients’ psychological states into structural equation modeling for analysis, thereby deepening our research into the factors influencing social alienation among cancer patients. (5) We did not perform stratified analysis based on diagnosis timing. Patients diagnosed at a later stage may face more frequent treatments and a heavier disease burden. These factors may exacerbate patients’ feelings of social alienation, thereby influencing study outcomes.

## 5 Conclusion

In summary, this study constructed a model of influencing factors of social alienation in cancer patients using SEM. By analyzing the effects of illness perception, fear of cancer progression, and perceived social support on social alienation, it clarified the pathways and magnitudes of relationships among variables. The findings of this study hold significant implications for the social care of cancer patients at all stages who meet the inclusion and exclusion criteria, providing valuable reference for developing interventions aimed at alleviating patients’ social alienation and improving their mental health. This model highlights the need for clinicians and nurses to focus on the determinants of social alienation in care, with targeted strategies to reduce alienation and enhance patients’ quality of life.

## Data Availability

The raw data supporting the conclusions of this article will be made available from the corresponding author upon request.

## Footnotes

1
https://www.danielsoper.com/statcalc/calculator.aspx?id=89
